# Hepatocyte ABCA1 deficiency is associated with reduced HDL sphingolipids

**DOI:** 10.3389/fphys.2023.1208719

**Published:** 2023-08-04

**Authors:** Alaa Othman, Mingxia Liu, Heiko Bode, Elena Boudyguina, Arnold von Eckardstein, John S. Parks, Thorsten Hornemann

**Affiliations:** ^1^ Institute of Clinical Chemistry, University Hospital Zurich and University Zurich, Zurich, Switzerland; ^2^ Department of Internal Medicine-Section on Molecular Medicine, Wake Forest University School of Medicine, Winston-Salem, NC, United States; ^3^ Department of Biochemistry, Wake Forest University School of Medicine, Winston-Salem, NC, United States

**Keywords:** sphingolipids, Tangier disease, serine-palmitoyltransferase, HDL, LDL, lipoprotein

## Abstract

ATP binding cassette transporter A1 (ABCA1) limits the formation of high density lipoproteins (HDL) as genetic loss of ABCA1 function causes virtual HDL deficiency in patients with Tangier disease. Mice with a hepatocyte-specific ABCA1 knockout (Abca1 HSKO) have 20% of wild type (WT) plasma HDL-cholesterol levels, suggesting a major contribution of hepatic ABCA1 to the HDL phenotype. Whether plasma sphingolipids are reduced in Tangier disease and to what extent hepatic ABCA1 contributes to plasma sphingolipid (SL) levels is unknown. Here, we report a drastic reduction of total SL levels in plasma of a Tangier patient with compound heterozygosity for mutations in ABCA1. Compared to mutation-free controls, heterozygous mutations in ABCA1 had no significant effect on total SLs in plasma; however, apoB-depleted plasma showed a reduction in total SL also in het carriers. Similarly, liver specific Abca1 KO mice (Abca1 HSKO) showed reduced total sphingolipids in plasma and liver. In parallel, apoM and sphingosine-1-phosphate (S1P) levels were reduced in plasma of Abca1 HSKO mice. Primary hepatocytes from Abca1 HSKO mice showed a modest, but significant reduction in total SLs concentration compared to WT hepatocytes, although SL *de novo* synthesis and secretion were slightly increased in Abca1 HSKO hepatocytes. We conclude that hepatic ABCA1 is a signficant contributor to maintaining total plasma pool of HDL sphingolipids, including sphingomyelins and S1P.

## Introduction

Sphingolipids (SL) are essential components of cellular membranes, but also serve as signaling molecules for a multitude of cellular events (Hait et al., 2006; Hla and Dannenberg, 2012). Sphingolipids in plasma and tissues consist of a great variety of subspecies. A structurally shared feature of all sphingolipids is the sphingoid base backbone that is typically formed by the conjugation of L-Serine with an acyl-CoA of variable length. The majority of sphingoid bases in human plasma consists of 18 carbons, although the length can vary between C16 and C20 (Lone et al., 2020). Sphingoid bases are either saturated (sphinganine, SA) or contain a single ∆4E (sphingosine, SO) or a combined ∆4E and ∆14Z double bond (Sphingadieniene, SAdianine). Under certain conditions, alanine instead of serine is conjugated forming a different class of 1-deoxySphingolipids (1-deoxySL) (Lone et al., 2019). Sphingoid bases are usually N-acylated with a second fatty acid and conjugated to a variety of O-linked head group structures forming a great spectrum of possible sphingolipid subspecies (e.g., ceramides, sphingolmyelins, hexosyl ceramides, gangliosides, etc.). Sphingomyelins (SM) are the major SLs in plasma and components of lipoproteins. Very low density lipoproteins (VLDL) and low density lipoproteins (LDL) contain greater proportions of SM than high density lipoproteins (HDL) due to proportionally higher VLDL and LDL concentrations compared to HDL (Chapman, 1986). However, SM is the second most abundant phospholipid and the most abundant sphingolipid in HDL (Martinez-Beamonte et al., 2013). SM regulates HDL metabolism by modulating the activity of lecithin: cholesterol acyltransferase (Subbaiah et al., 2012) and phospholipid transfer protein (Sweeny and Jonas, 1985) as well as ABCA1-mediated cellular cholesterol efflux (Takahashi et al., 2006; Worgall, 2011) and SR-BI-mediated removal of HDL cholesterol (Yancey et al., 2000). In addition, sphingosine 1-phosphate (S1P), a prominent sphingolipid signaling molecule, is enriched in HDL as it specifically binds to apoM (65%) (Aoki et al., 2005). However, compared to cholesterol, triglycerides and phosphatidylcholines, the metabolic origin and fate of sphingolipids carried by HDL is little understood.

ATP binding cassette transporter A1 (ABCA1) is a membrane transporter that transports free cholesterol, phosphatidylcholine (PC), and SM to lipid-free apoA-I, forming nascent HDL particles (Lee and Parks, 2005; Yokoyama, 2005). Nascent HDL particles generated by non-hepatic, ABCA1-expressing HEK293 cells contain ∼10% SM regardless of particle size (Sorci-Thomas et al., 2012). ApoB lipoprotein-depleted plasma from humans with heterozygous ABCA1 mutations had ∼34% and 12% reduction in S1P and apoM, respectively; however, plasma SM concentration was not examined (Karuna et al., 2011). Although our previous studies in hepatocyte-specific ABCA1 knockout (Abca1 HSKO) mice suggested that hepatic ABCA1 activity accounts for ∼80% of normal plasma HDL-Cholesterol (Timmins et al., 2005), the contribution of hepatic ABCA1 to plasma sphingolipids concentrations is unknown.

Here we used Abca1 HSKO mice to determine to what extent the loss of ABCA1 affects plasma sphingolipid concentrations and hepatic sphingolipid production. Results were compared to plasma samples from individuals with homo and heterozygous loss of function mutations in ABCA1.

## Materials and methods

### Human samples

Six subjects with heterozygous mutations in ABCA1, one compound heterozygote ABCA1 and eight unaffected members of a Dutch family were studied. Subject description, sample collection, ethics approval and plasma lipids/apolipoprotein quantification have been reported previously (Karuna et al., 2010; Karuna et al., 2011). ApoB-depleted human plasma was generated by precipitation with dextran sulfate/Mg^2+^ as described previously (Karuna et al., 2011).

### Animals

Abca1 HSKO mice were generated as described previously (Timmins et al., 2005). Mice were housed in the Wake Forest University School of Medicine animal facility with a 12 h light/12 h dark cycle and fed a commercial chow diet *ad libitum*. All animal procedures were approved by the Institutional Animal Care and Use Committee of Wake Forest University School of Medicine. Abca1 HSKO and Abca1 fl/flWT littermates (hereafter referred to as WT) were derived from heterozygous mating. Seventeen week-old mice were sacrificed after a 4 h fast or in the non-fasting state.

### S1P analysis

Mouse plasma S1P were analysed as described previously (Karuna et al., 2011).

### Primary hepatocyte isolation

Primary hepatocytes were isolated as described previously (Chung et al., 2010) with minor modifications. After isolation from liver, hepatocytes were centrifuged at 50 × g for 5 min in a 50% Percoll-containing Williams’ media E to pellet live cells, which were then washed with Williams’ media E before seeding in 35 mm dishes at a density of 0.4 × 10^6^ cells per dish.

### Stable isotope labeling

Hepatocytes were cultured for 2 h in Williams’ E media and then switched to MEM media for 2 h. For labelling hepatocytes were incubated with MEM for 24 h containing 1 mM d3-serine and 2 mM C13-alanine (Cambridge Isotope Laboratories) with or without myriocin (1 μg/mL in MetOH, Sigma). Cells were pelleted and lysed with extraction buffer (50 mM HEPES, pH 7.4, 1 mM EDTA, 0.2% Triton X100) and protein concentrations measured by BCA. 90 µg of protein in 100 µL of extraction buffer were used for lipid extraction and sphingoid base analysis.

### Sphingoid bases analysis

The sphingoid base profile was analysed as described before (Othman et al., 2012). Tissues were homogenized in PBS [pH 7.4) with 0.2% Triton X-100 (vol/vol)] using a Precellys 24 tissue homogenizer (Bertin Technologies). 100 µL plasma, 80 µg tissue homogenate or 90 μg cell lysates were extracted for lipid analysis. Methanol (0.5 mL) spiked with 200 pmol of the internal standards d7-sphingosine and d7-sphinganine (d7SA, d7SO; Avanti Polar Lipids) were added to the aliquot of plasma or tissue homogenate.

The sphingoid bases were separated on a C_18_ column (Uptispere 120 Å, 5 µm, 125 mm × 2 mm, Interchim) and analysed on a TSQ Quantum Ultra MS (Thermo) as described before (Mwinyi et al., 2017; Lone et al., 2020). Intra- and inter-assay variation (CV %) of the method was between 5% and 20%.

14 different sphingoid base backbones were analysed (C_16_SO, C_16_SA, C_17_SO, C_17_SA, C_18_SO, C_18_SA, C_18_SAdiene, C_19_SO, C_20_SO, C_20_SA, deoxysphinganine (1-deoxySA), deoxysphingosine (1-deoxySO), deoxymethylsphinganine (doxmethSA), deoxymethylsphingosine (doxmethSO) sphingoid bases were reported.

### Western blotting

Plasma from WT and Abca1 HSKO mice was subjected to a 12% SDS-PAGE and transferred to a nitrocellulose (Schleicher and Schuell). Membranes were blocked with 5% milk in TBST buffer, incubated with primary antibody at 4°C overnight, washed three times, and then incubated with secondary antibody for 1 h at room temperature. Blots were exposed using Supersignal West Pico chemiluminescence substrate (Pierce) and visualized with a Fujifilm LAS-3000 camera. Antibodies for Western blots included: anti-mouse apoM antibody (LifeSpan), anti-mouse apoA-I primary antibody (Biodesign), and anti-rabbit secondary antibody (GE Healthcare). Quantification of band intensity was performed using Multi Gauge software.

### Statistical analysis

Two-tailed Student’s *t*-test was used to compare results for human ABCA1 mutations (normal vs. heterozygotes) and mouse genotypes (WT vs. Abca1 HSKO). *p*-value < 0.05 was considered statistically significant.

## Results

In this study, we investigated the change in total plasma sphingolipid levels in response to hepatic ABCA1 activity. Plasma SL’s consist of hundreds structurally different subspecies because of the heterogeneity in conjugated alkyl chains and head group structures. To reduce this complexity, the extracted plasma SLs were subjected to a sequential acid and base hydrolysis prior to analysis. This hydrolysis step removes the N-linked alkyl chain and O-linked head group structures and results in the release of the free sphingoid base backbones, which are the common structural element of all SL (Bertea et al., 2010). The sum of all individual sphingoid bases, therefore, reflects the total sphingolipid content in the analyzed sample.

### Humans with mutations in ABCA1 have decreased HDL sphingolipid concentrations

**FIGURE 1:**
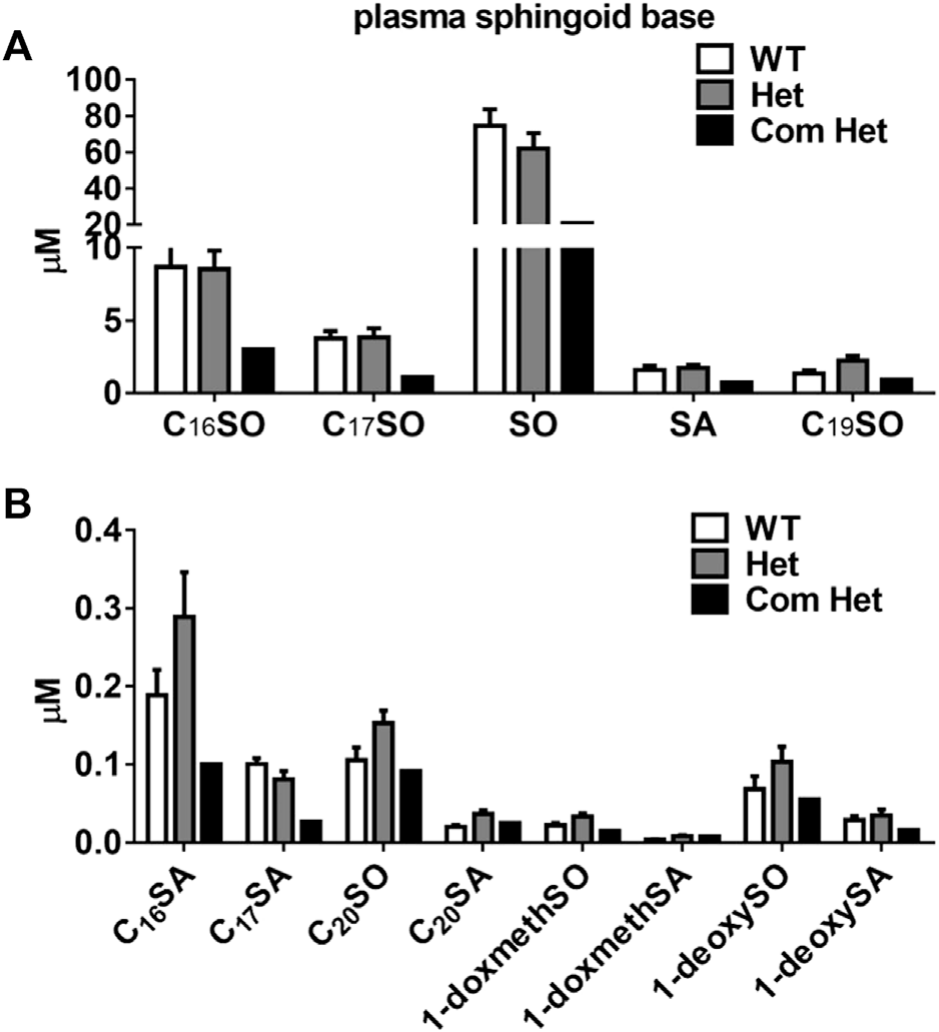
Plasma sphingoid base concentrations in humans with ABCA1 mutations. Sphingoid base profile in subjects with heterozygous mutations in ABCA1 (Het, *n* = 8), their family controls (WT, *n* = 6) and a compound heterozygous subject (Com Het, *n* = 1) **(A)** C_16_SO, C_17_SO, SO, SA, C_19_SO and **(B)** C_16_SA, C_17_SA, C_20_SO, C_20_SA, doxSA, doxSO, doxmethSA and doxmethSO were quantified. SO, sphingosine; SA, sphinganine; doxSA, deoxysphinganine; doxSO, deoxysphingosine; doxmethSA, deoxymethylsphinganine; doxmethSO, deoxymethylsphingosine. Data are presented as mean ± SEM. There were no statistically significant differences between WT and Het subjects by Student’s *t*-test.

**FIGURE 2:**
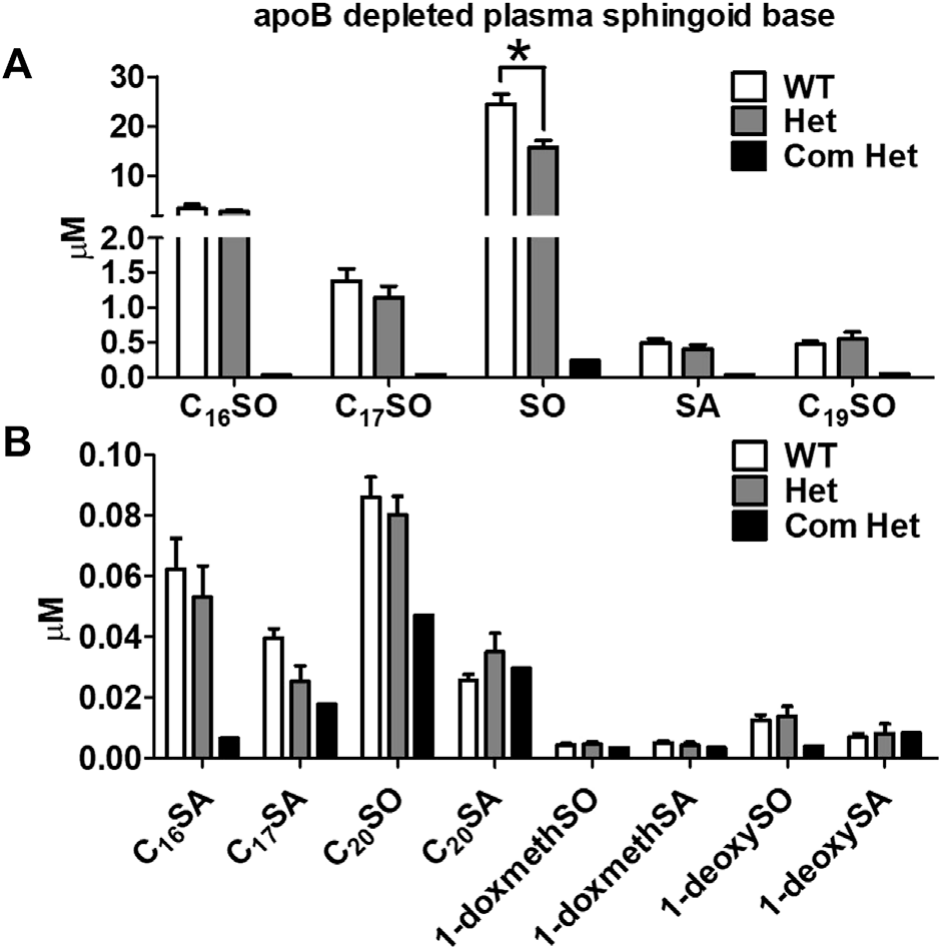
Decreased plasma HDL sphingoid base concentrations in subjects with ABCA1 mutations. Sphingoid base profile in ApoB-depleted plasma from subjects with heterozygous mutations in ABCA1 (Het, *n* = 8), their family controls (WT, *n* = 6), and a compound heterozygous subject (Com Het, *n* = 1) **(A)** C_16_SO, C_17_SO, SO, SA, C_19_SO and **(B)** C_16_SA, C_17_SA, C_20_SO, C_20_SA, doxSA, doxSO, doxmethSA and doxmethSO were quantified. Data are presented as mean ± SEM, *p*-values were calculate by two-tailed Student’s *t*-test, *p < 0.05, **p < 0.01.

The plasma of a single Tangier patient with compound heterozygous mutations in ABCA1 showed a drastic reduction in all plasma sphingoid bases. The relative decrease was about 70% for the most abundant sphingoid bases (C18SO and C16SO), but less pronounced for the minor species. In contrast, heterozygous (Het) mutations in ABCA1 show reduced plasma HDL-cholesterol, apoA-I, and S1P compared to their family controls (WT) (Karuna et al., 2010; Karuna et al., 2011), but did not show a significant difference in total plasma SL levels compared to the unaffected family members (Figures 1A, B). However, in apoB-depleted plasma, which lacks VLDL and LDL but still contains HDL and albumin, C18SO and C16SO were almost absent in the Tangier patient plasma and C18SO was also significantly reduced in the Het carriers (Figures 2A, B). Collectively, our data suggest a decrease in HDL sphingolipids in humans with ABCA1 mutations, indicating that ABCA1 activity determines sphingolipid levels, most likely by maintaining HDL particle concentration.

### Abca1 HSKO mice have decreased plasma sphingolipid concentrations

**FIGURE 3:**
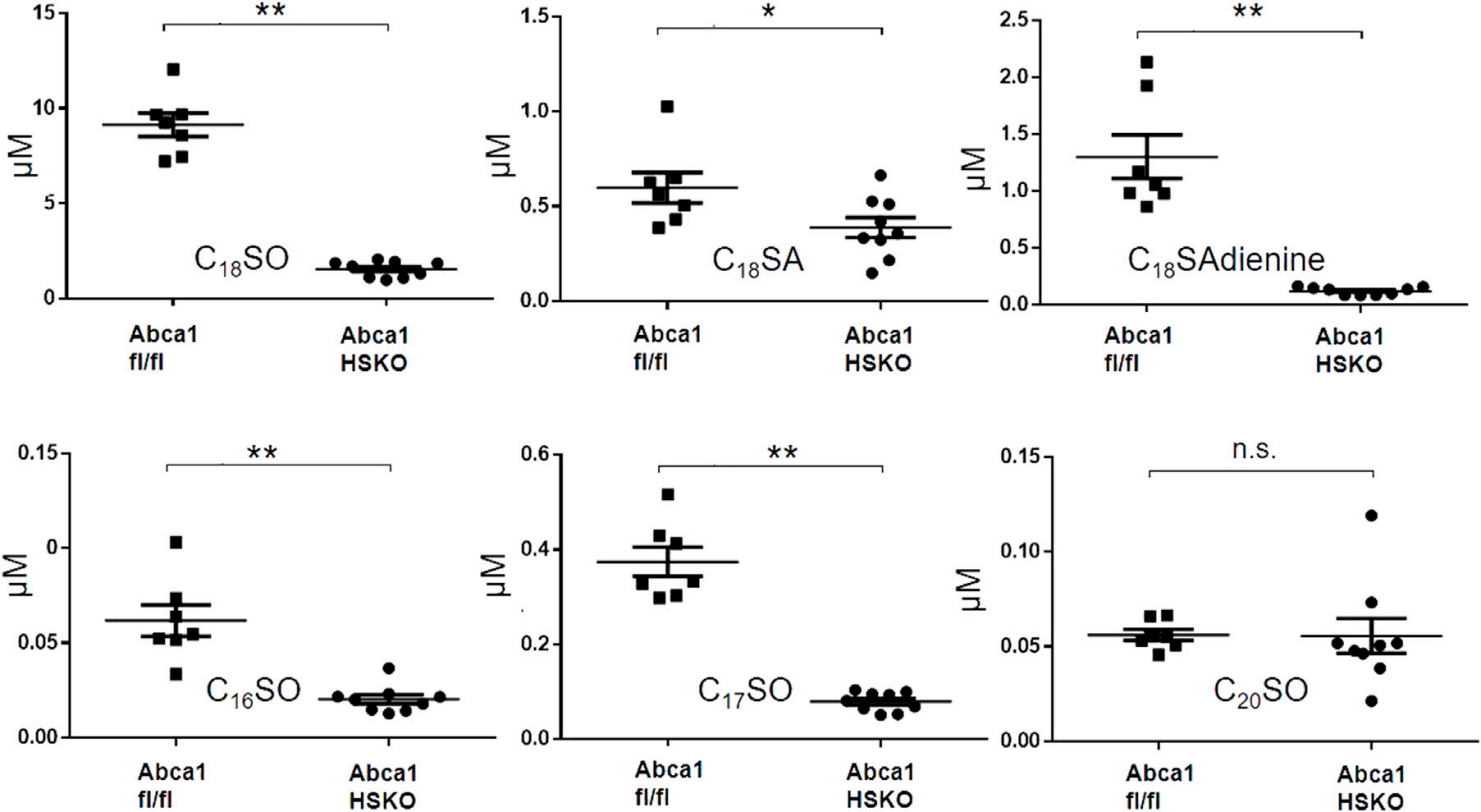
Decreased plasma sphingoid base concentrations in Abca1 HSKO mice. Sphingoid base profile in plasma of WT and Abca1 HSKO mice. C_18_SO, C_18_SA C_18_SAdiene C_16_SO, C_17_SO and C_20_SO sphingoid bases were quantified. Data are presented as mean ± SD. *p*-values were calculate by two-tailed Student’s *t*-test, *p < 0.05, **p < 0.01.

We previously reported that hepatic ABCA1 activity contributes ∼80% of the plasma HDL-cholesterol pool in chow-fed mice (Timmins et al., 2005). To determine whether hepatocyte ABCA1 is also a major contributor to plasma HDL sphingolipids, we compared the sphingoid base profile between Abca1 HSKO mice and WT littermate controls (Abca1 fl/fl). As shown in Figure 3, there was a significant decrease in plasma levels of C_18_SO, C_18_SA, C_18_SAdiene C_16_SO, and C_17_SO but not of C_20_SO in Abca1 HSKO mice Because plasma triglyceride concentrations are elevated in Tangier disease subjects (Clee et al., 2000; Kolovou et al., 2003) and non-fasted Abca1 HSKO mice (Chung et al., 2010), we performed a similar sphingoid base profiling experiment with non-fasted Abca1 HSKO mouse plasma, which gave similar results (Supplementary Figure S1). Collectively, our data showed that hepatocyte ABCA1 deficiency leads to significantly reduced HDL plasma sphingolipid levels.

### Abca1 HSKO mice have decreased hepatic sphingolipid levels

**FIGURE 4:**
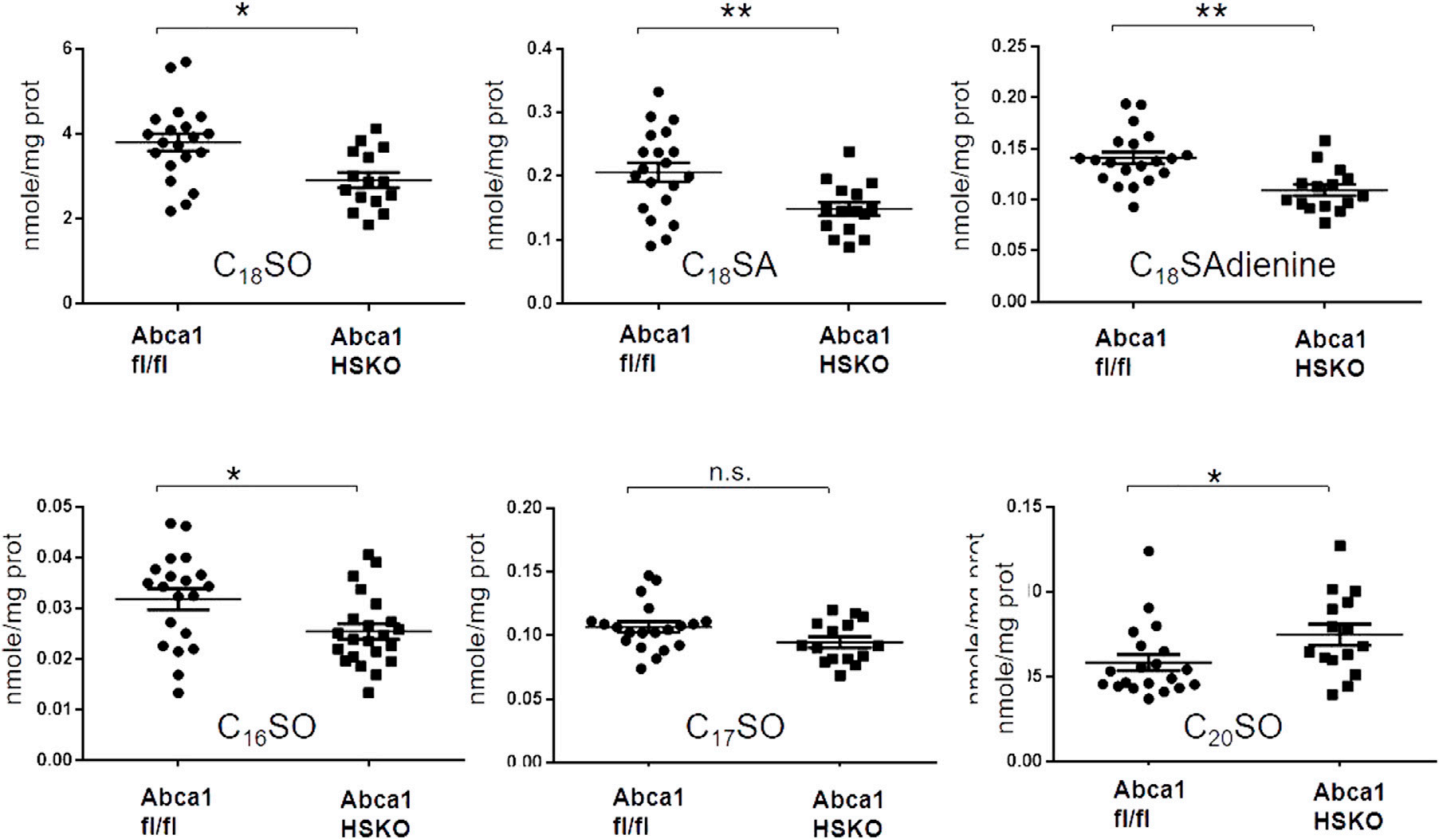
Decreased liver sphingoid base content in Abca1 HSKO mice. Sphingoid base profile in liver lysates from WT and Abca1 HSKO mice C_18_SO, C_18_SA C_18_SAdiene C_16_SO, C_17_SO and C_20_SO sphingoid bases were quantified. Data are presented as mean ± SD. *p*-values were calculated by two-tailed Student’s *t*-test, *p < 0.05, **p < 0.01.

We next asked whether the lack of HDL as a lipid transporter is the primary cause for the reduction in plasma sphingolipids or whether ABCA1 deficiency in Abca1 HSKO is also associated with lower cellular levels and an altered SL synthesis. To address these questions, we analyzed total SL levels in hepatic tissue of the mice and found a significant reduction in the major (C_16_SO, C_18_SO, S_18_SA, and C_18_SAdiene), but not in the minor sphingoid bases (C_17_SO, C_19_SO, C_20_SO, C_20_SA, and doxSA) (Figure 4).

### *De novo* SL formation and secretion is increased in primary Abca1 HSKO hepatocytes

**FIGURE 5:**
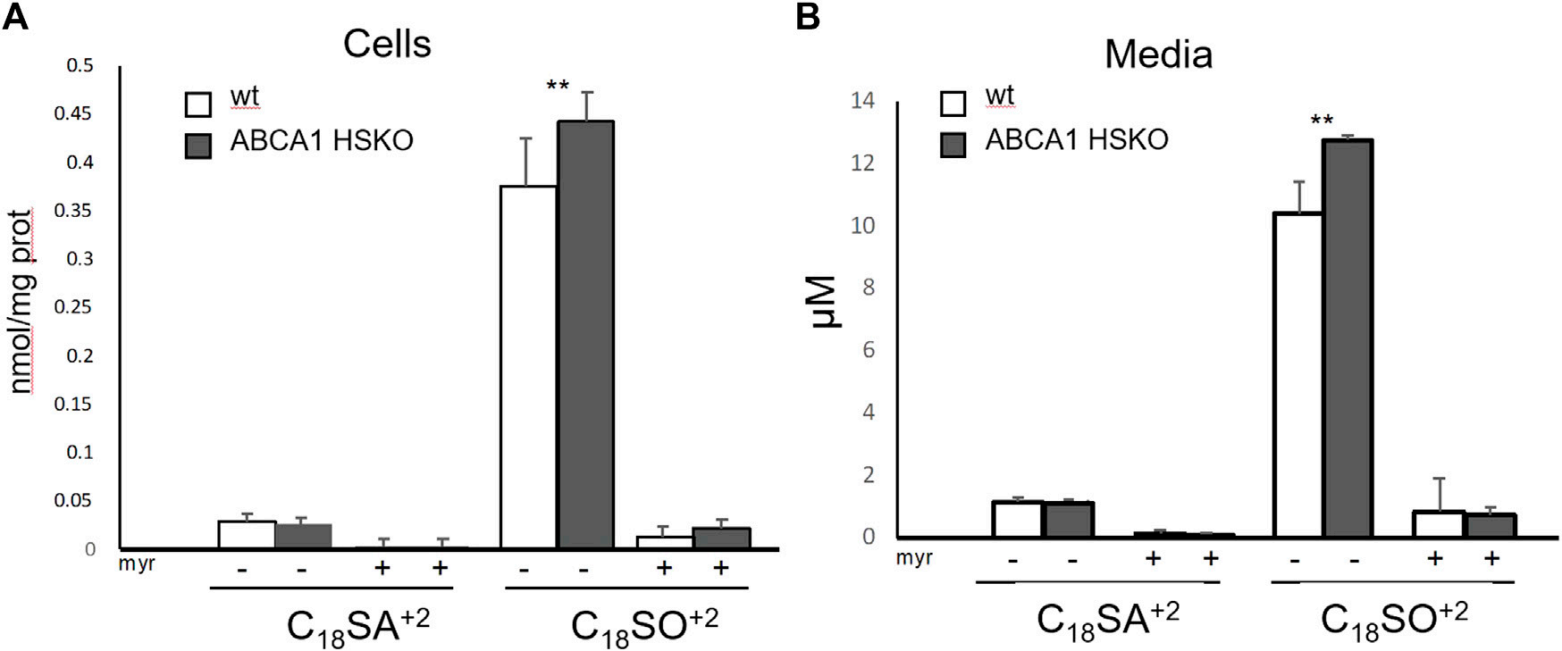
Increased secretion of newly synthesized sphingolipids in Abca1 HSKO mouse hepatocytes. Hepatocytes from WT and Abca1 HSKO mice were isolated and incubated with d3-serine in presence of vehicle (MeOH) or myriocin for 24 h. Cells and media were collected and subjected to a sphingoid base analysis. Newly synthesized sphingoid bases in cells **(A)** and media **(B)** were quantified. *n* = 4 per group. Data are presented as mean ± SD Two-way ANOVA was used for statistical analysis. *p < 0.05, **p < 0.01.

To determine whether sphingolipid synthesis is altered in hepatocytes lacking ABCA1, we measured the *de novo* SL synthesis in cultured primary hepatocytes by the incorporation of stable isotope labelled d3N15-serine into *de novo* formed sphingoid bases. Myriocin (myr), a specific inhibitor of sphingolipid *de novo* synthesis was added as negative control. Surprisingly, the total amount of newly synthesized SLs was slightly higher in Abca1 HSKO hepatocytes (Figure 5A) compared to WT controls. A similar picture was seen when analyzing the export of the labelled SL into the media, which was also higher for SO by Abca1 HSKO hepatocytes (Figure 5B). This was specific as Myriocin completely blocked sphingolipid *de novo* synthesis in both wild type and Abca1 HSKO cells.

### Hepatocyte ABCA1 deficiency results in reduced plasma S1P and apoM

**FIGURE 6:**
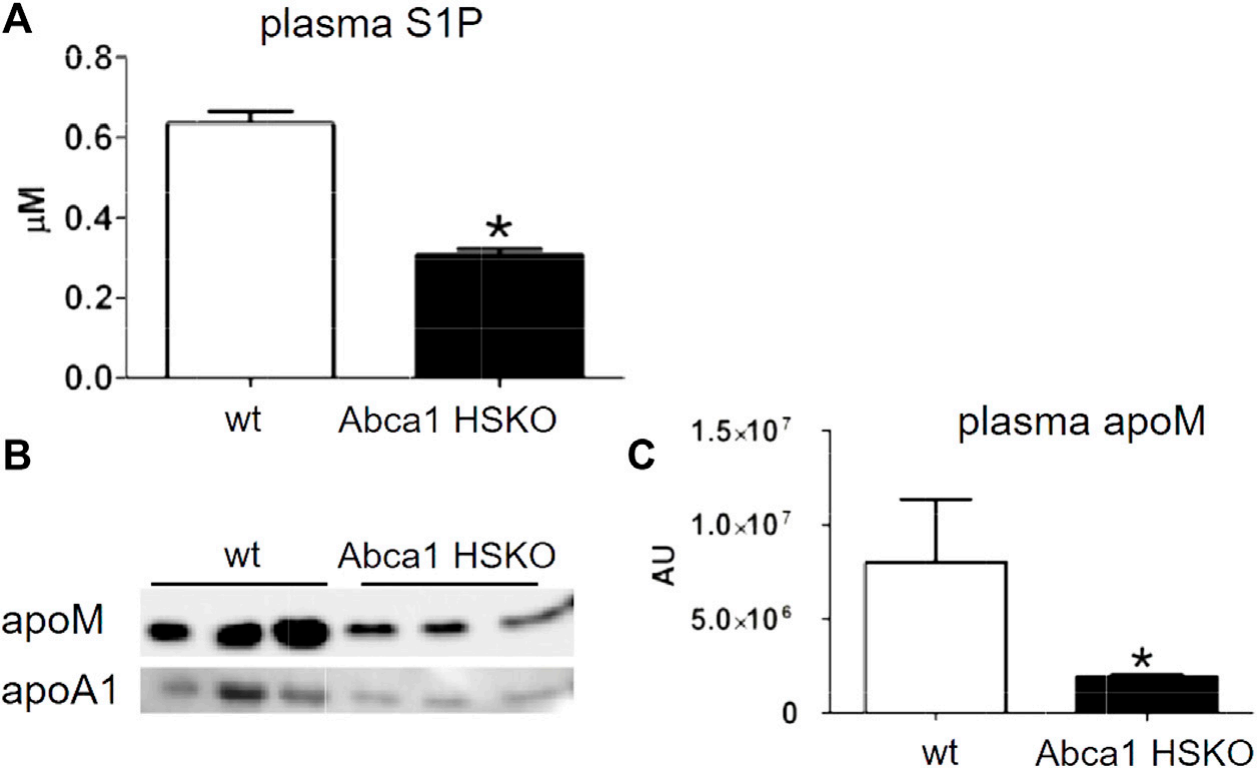
Decreased plasma S1P and apoM in Abca1 HSKO mive. **(A)** Plasma S1P levels were analyzed as described previously (Karuna et al., 2011) for WT (*n* = 26) and Abca1 HSKO (*n* = 21) mice. *p < 0.05. **(B)** WT and Abca1 HSKO mouse plasma was analysed by Western blot for mouse apoM and apoA-I. **(C)** ApoM band intensity was quantified, plotted, and analyzed. Data were log transformed and analyzed by two-tailed Student’s *t*-test. *p < 0.05, **p < 0.01.

About 60% of the plasma S1P is transported by HDL and about 30% bound to albumin. The binding to HDL is mediated by apoM (Christoffersen et al., 2011). Previously, we reported reduced plasma S1P in subjects with ABCA1 mutations (Karuna et al., 2011). Thus, we analyzed plasma S1P and apoM levels in Abca1 HSKO mice. Both, S1P and apoM concentrations were significantly lower in plasma of Abca1 HSKO mice (Figures 6A–C).

## Discussion

Here, we show that total plasma sphingolipids were greatly reduced in patients with Tangier disease and apoB-lipoprotein free plasma of individuals with compound heterozygous ABCA1 mutations. A similar SL-deficient phenotype was confirmed in plasma of Abca1 HSKO mice compared to WT mice. The biggest difference was seen for SLs with a C18SO backbone. SM is the most abundant SL in plasma and it was already shown previously, that total SM concentrations are reduced in plasma and HDL of Tangier patients (Yao et al., 1978; Frohlich and Godin, 1986). However, the reduction in total plasma SL in ABCA1 deficient humans was less pronounced compared to Abca1 HSKO mice, likely due to the higher proportion of VLDL and LDL in humans which transport two-thirds of the total plasma sphingomyelin (Nilsson and Duan, 2006). Isolated low levels of SM in the blood may not necessarily lead to specific consequences or symptoms on their own. However, SM is important for neuron and glia formation. Demyelinating polyneuropathy is frequently encountered in the in Tangier patients but reasons are not yet fully understood. The genotype-phenotype correlation is not currently straightforward because different phenotypic characteristics. However, besides the impaired cholesterol metabolism in Tangier patients also reduced levels of plasma SM might contribute to these neurological effects.

Several mechanisms might explain the reduced HDL sphingolipid content in the absence of ABCA1. First, ABCA1 mediates the efflux of SM, PC, and FC to form nascent HDLs (Sorci-Thomas et al., 2012). Less SM efflux from ABCA1-deficient hepatocytes could partially contribute to a reduced HDL SM content. Second, HDL particles are hyper-catabolized in Tangier patients as well as Abca1 HSKO mice (Timmins et al., 2005). SL levels in HDL might therefore be low due to joint removal or due to the reduced number of particles that can elicit SM efflux of SM via ABCG1 or other transporters (Kobayashi et al., 2006). Third, phospholipids like SM may be transferred from VLDL and LDL to HDL by the activity of the phospholipid transfer protein (PLTP) (Jiang et al., 1999). Plasma PLTP activity was reduced ∼80% in whole body ABCA1 knockout mice (Francone et al., 2003) and completely eliminated in Abca1 HSKO mice (Timmins et al., 2005), suggesting defective transfer of SM from VLDL or LDL to HDL due to the very low levels of both PLTP and HDL acceptor particles. Fourth, hepatic sphingolipid synthesis and secretion might be reduced in Abca1 HSKO mice. To test the latter hypothesis, we analyzed the SL content of livers in Abca1 HSKO mice as well as synthesis and secretion of SL by cultured Abca1 HSKO hepatocytes. In the liver of Abca1 HSKO mice, we found a reduction of the major sphingoid bases, although to a lower extent as in plasma. Surprisingly, analyzing the *de novo* synthesis and export of SL in cultured primary hepatocyte using a stable isotope labelling approach revealed a modest, but significant, increase in the formation and secretion of C18SO in hepatocytes from Abca1 HSKO mice. However, cellular SL synthesis is regulated by a homeostatic feedback mechanism, which is controlled by local ceramide levels in the ER. The majority of SL in the cell are sphingomyelins, which are abundant in the plasma membrane. Is possible that a reduction in total SM stimulates SL *de-novo* synthesis which is reflected by increase in labelled +2 sphingoid bass (Figure 5). As the isotope labeled +2 species are not included in the total SL analysis (Figure 4) it might explain the opposing results between increased *de-novo* formed and reduced total levels.

### Hepatic ABCA1 and S1P

We previously reported a significant reduction in plasma S1P and apoM in apoB-depleted plasma (i.e., HDL) of humans with heterozygous ABCA1 mutations (Karuna et al., 2011). In agreement with the human data, we also found significant reductions in plasma S1P and apoM levels in Abca1 HSKO mice, suggesting that hepatocyte ABCA1 is a major contributor to plasma HDL-S1P levels. Liver and hepatocytes can produce S1P (Kurano et al., 2013) but whether hepatocyte ABCA1 is directly involved in S1P transport from liver to plasma is unknown. It was suggested earlier that ABCA1 mediates S1P transport from erythrocytes (Kobayashi et al., 2009), which are the major source for plasma S1P (Pappu et al., 2007) and express significant amounts of ABCA1 (Ohkawa et al., 2020).

HDL stimulates secretion of apoM (Ahnstrom et al., 2010), which is the major carrier for plasma S1P (Christoffersen et al., 2011). Thus, plasma HDL reduction due to ABCA1 deficiency results in less plasma apoM to transport S1P. Regardless, ABCA1 regulation of apoM and S1P merits further investigation considering the atheroprotective role of apoM/S1P-containing HDL (Egom et al., 2013).

Abca1 HSKO mice had attenuated PI3 kinase activation in liver in response to insulin, but the mechanism is still unclear (Chung et al., 2010). S1P activates PI3 kinases in liver and hepatocytes (Osawa et al., 2010; Studer et al., 2012), suggesting that attenuated PI3 kinase activation in Abca1 HSKO mice may be partially due to reduced plasma and HDL-S1P (Key et al., 2017).

In conclusion, our results demonstrate that hepatocyte ABCA1 is a major contributor in maintaining normal plasma sphingolipids, including SM and S1P. However, further research is necessary to find out whether the altered SL metabolism is a direct consequence of ABCA1 deficiency or secondary to HDL deficiency caused by the lack of ABCA1.

## Data Availability

The raw data supporting the conclusion of this article will be made available by the authors, without undue restrictions.

## Supplementary material

SUPPLEMENTARY FIGURE S1 Decreased plasma sphingolipid concentrations in non-fasted Abca1 HSKO mice. Plasma sphingoid base profile in non-fasting WT and Abca1 HSKO mice **(A)** C_17_SO, C_18_SO, C_18_SA and C_18_SAdiene and **(B)** C_16_SO, C_19_SO, C_20_SO, C_20_SA and doxSA were quantified. Data are presented as mean ± SEM. WT (*n* = 7) HSKO (*n* = 9), Student’s *t*-test, **p* < 0.05, ** *p* <0.01.
