# Supplementary material for: Hepatocyte ABCA1 deficiency is associated with reduced HDL sphingolipids
Source: Front Physiol. 2023 Aug 4;14:1208719. doi: 10.3389/fphys.2023.1208719 (PMC10436503; doi:10.3389/fphys.2023.1208719)
Supplement: Supplementary file 1 [file Image1.pdf]

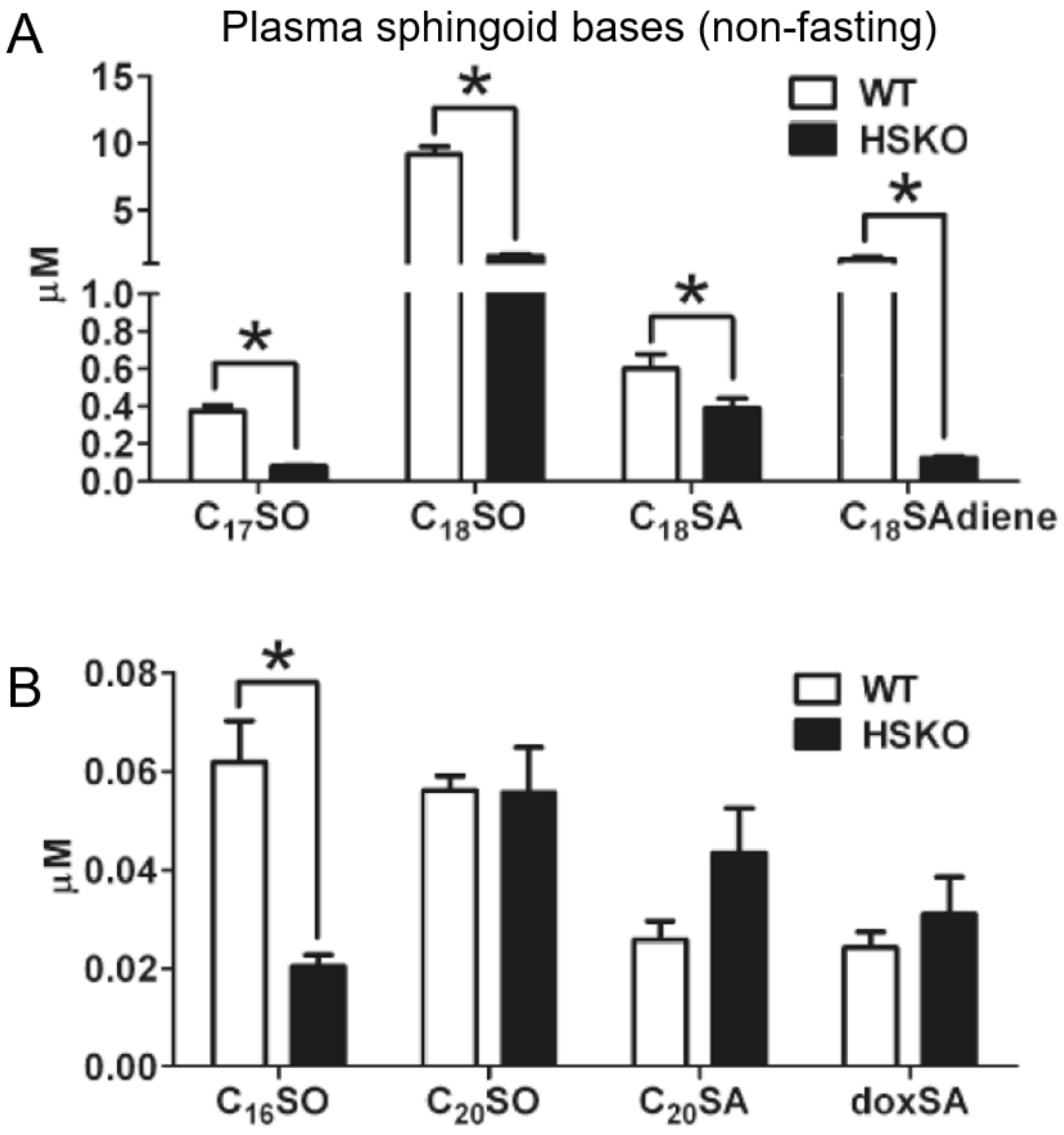

Supplemental Figure 1. Decreased plasma sphingolipid concentrations in non-fasted Abca1 HSKO mice

Plasma sphingoid base profile in non-fasting WT and Abca1 HSKO mice (**A**) C_{17}SO, C_{18}SO, C_{18}SA and C_{18}SAdiene and (**B**) C_{16}SO, C_{19}SO, C_{20}SO, C_{20}SA and doxSA were quantified. Data are presented as mean ± SEM. WT (n=7) HSKO (n=9), Student t test, *p < 0.05, ** p<0.01
